# Data management in the digital health environment scale development study*

**DOI:** 10.1186/s12913-023-10205-3

**Published:** 2023-11-14

**Authors:** Hasan Fehmi Demirci, Elif Dikmetaş Yardan

**Affiliations:** Ondokuz Mayıs University, Health Sciences Faculty Department of Healthcare Management, OMÜ Kurupelit Campus, Samsun, Türkiye Atakum 55200

**Keywords:** Digital, Health, Data management, Measurement tool, Factor analysis, Scale development

## Abstract

**Purpose:**

This study aims to develop a scale that measures individuals' perceptions of privacy, security, use, sharing, benefit and satisfaction in the digital health environment.

**Method:**

Within the scope of the study, in the scale development process; The stages of literature review, creation of items, getting expert opinion, conducting a pilot study, ensuring construct and criterion validity, and reliability analyses were carried out. The literature was searched for the formation of the question items. To evaluate the created question items, expert opinion was taken, and the question items were arranged according to the feedback from the experts. In line with the study's purpose and objectives, the focus group consisted of individuals aged 18 and above within the community. The convenience sampling method was employed for sample selection. Data were collected using an online survey conducted through Google Forms. Before commencing the survey, participants were briefed on the research's content. A pilot study was conducted with 30 participants, and as a result of the feedback from the participants, eliminations were made in the question items and the scale was made ready for application. The research was conducted by reference to 812 participants in the community. Expert evaluations of the question items were obtained, and a pilot study was conducted. A sociodemographic information form, a scale developed by the researcher, Norman and Skinner's e-Health Literacy Scale, and the Mobile Health and Personal Health Record Management Scale were used as data collection tools. Results: The content validity of the research was carried out by taking expert opinions and conducting a pilot study. Exploratory factor analysis and confirmatory factor analysis were performed to ensure construct validity. The total variance explained by the scale was 60.43%. The results of confirmatory factor analysis indicated that the 20-Item 5-factor structure exhibited good fit values. According to the analysis of criterion validity, there are significant positive correlations among the Data Management in the Digital Health Environment Scale, Norman and Skinner’s e-Health Literacy Scale and the Mobile Health and Personal Health Record Management Scale (*p* < 0.01; *r* = .669, .378). The Cronbach's alpha value of the scale is .856, and the test–retest reliability coefficient is .909. Conclusion: The Data Management in the Digital Health Environment Scale is a valid and reliable measurement tool that measures individuals' perceptions of privacy, security, use, sharing, benefit and satisfaction in the digital health environment.

## Introduction

The digitalization process has been enhanced worldwide over the last twenty years. The widespread use of the internet in all sectors has provoked digitalization, which has led to various innovations. All these innovations have enabled the health sector to achieve its share of digital transformation. In addition to the innovations in the Internet and healthcare sectors, various factors have facilitated the proliferation of digital healthcare services. These factors include technological advancements, the centralization of patients in healthcare services, the increasing aging population, and easy access to information at any time and place. As a result of these factors, emerging digital healthcare services offer numerous benefits to service providers and patients. In this respect, digital health services include collaborative or interactive applications of modern information and communication technologies that enable the provision of health services and the improvement of public health [1]. In this context, digital health technologies are a helpful tool to increase access to health services, efficiency, accountability, and resilience of health systems [2].

The use of digital systems to meet the demands and expectations of people, especially with respect to their health, is inevitable in health services, in which the patient is centred. Developments in health informatics, sensor technologies and mobile devices have made it easier for people to access health services. At the centre of the use of digital systems in health services is the idea of ​​"leading a healthy, high quality and prosperous life" [3]. Considering these circumstances, digital technology has made new and innovative contributions to healthcare services [4].

Information and communication technologies are among the prominent components of health systems. Researchers have claimed that the advances in digital technologies and data science made in recent years will have a tremendous impact on health services, and it has been widely predicted that these tendencies orient contemporary health services towards digital health [5]. Therefore, the phenomenon of digital health has emerged as an important dimension of contemporary health policy and delivery in many countries [6].

### Digital health

Digital health is defined as “the use of information and communication technologies to improve human health, health services, and the quality of healthy life for individuals and societies” [7]. The use and scaling of digital health solutions allow people worldwide to maintain higher health standards, promote their health and well-being, and access health services more easily, thus protecting the health of individuals [8]. Digital health focuses on connecting the systems, tools, medical devices, and services that provide essential health care, thereby providing critical data insights that were not previously available to all stakeholders in the field of health care delivery [9].

### Digital health environment

With the utilization of digital health solutions, a new digital health environment has emerged, characterized by the presence of numerous health-related information tailored to individuals, built upon information and communication technology. The digital health environment refers to the environment in which the information and resources obtained through the provision of health services are kept, shared, and managed on digital platforms and is among the new trends that have emerged in the field of health in recent years. Recent trends in healthcare delivery encourage integrated and patient-centered care provided by professionals during the course of a disease [10]. From this perspective, the digital health environment can be considered as an approach that facilitates patient-centered care. Digital health environment is often used as a broad umbrella term that includes mobile health (m-health) as well as emerging areas such as electronic health (e-health) and the use of advanced computer science in the fields of big data and artificial intelligence [11]. Digital health environment is concerned with the task of improving human health using high-profile applications such as wearable and implantable technology, web, email, mobile technology, social networking, data management and analytics [12]. Digital health environment encompasses a wide range of new digital technologies related to health. Such technologies are based on recent advances in the collection and analysis of an ever-increasing amount of data from both patients and healthy citizens [13]. In the contemporary world, digital technologies provide more security opportunities than paper-based records as a result of electronic health records. Laboratory reports for the patient, details of hospital stay and information about prescriptions can be archived, thus providing easy access to information [3].

Digital health environment aimed at a wide variety of purposes are under development [14]. Digital health environment, which facilitate the collection and sharing of data for patients, consist of many other systems. Those systems, which can be used instead of traditional systems, facilitate direct communication between the health professional and the patient. However, researchers have claimed that digital health systems that offer new opportunities may lead to many ethical problems [13].

### Privacy and security

Digital systems that contain large amounts of data regarding individuals entail security concerns and the problem of the ability of unauthorized persons to access the stored data through attacks, neglect or abuse. Another issue is privacy concerns. Digital technologies may violate some basic principles of information security and privacy due to unregulated access to stored information and personal data [15]. Confidentiality is a major concern regarding medical information [16].

The privacy and security breaches that occur in digital systems do not occur only for institutional and systemic reasons. A user installs a health app on their smart device. The user's data are collected on the device. The processing and storage of the data take place on their device. The data are transferred to the provider of the application, usually for the purposes of processing and storage. Therefore, misuse of the data through manipulation may lead to serious consequences for the user [17]. Digital interactions, such as using mobile apps, searching the internet, or chatting on social media, often generate health-related information [18]. Ensuring the security of this information requires individual precautions. Unless such precautions are taken, the information retained in the digital environment will inevitably be stolen and misused by unauthorized persons. Therefore, individuals need to know which personal information is kept in the digital health environment and how to manage this data.

### Data management in the digital health environment

Data management in digital health can be defined as people's ability to manage their own health information and data, to ensure the confidentiality and security of this data, and to request health services effectively using this data. Data management in the digital health environment requires at least a basic and conceptual knowledge of data collection, storage and normalization [19]. The digital health environment is a tool to improve health and healthcare delivery by ensuring effectiveness, efficiency, accessibility, safety, and personalization [20]. Concepts that can be evaluated in the digital health environment include digital literacy, digital self-efficacy, technology access, and attitudes toward use [21]. Today, where the internet is used extensively, the use of technologies related to the digital health environment has become the basis of health service delivery. It is stated that a positive attitude towards the digital health environment can improve health literacy, increase patients' participation in health services, and enable patients to better manage their health [22].

Recently, the national and international literature on digital health has emphasized concepts such as privacy, security, benefit satisfaction, ease of use, accessibility and satisfaction. Among the main gaps defined by the World Health Organization regarding digital health are the knowledge and attitudes of individuals and their behaviors towards digital health [23]. In this context this study aimed to develop a "Data Management in the Digital Health Environment Scale " to compensate for the limited number of studies that have been conducted to identify individuals’ perceptions regarding the health data stored in the digital environment and the limited number of measurement tools used to measure the perceptions of individuals regarding patient data in the digital environment. With the developed scale, people's positive attitudes towards their digital health information are important in terms of improving health decisions and adopting a health lifestyle. In addition, with the scale, it can be determined what the privacy and security perceptions of the health information kept in the digital environment are. Therefore, the scale developed in this study is important to determine the attitudes of individuals towards their own health information.

### Importance of the research

Due to the development of technology, digital technologies are used more intensively in the contemporary health system. In addition, individuals have started to benefit more from the opportunities offered by digital health through the use of various mobile technologies. For this reason, it is important to identify the perceptions of individuals regarding these technologies and applications as well as their concerns about privacy and security.

### Purpose of the research

This research aims to develop a new measurement tool to measure the privacy, security, use, sharing, benefit and satisfaction perceptions of individuals regarding their data in the digital health environment.

## Methods

### Study design

The research is a cross-sectional scale development study. The following processes suggested by Devellis (2021) for scale development were applied [24]:

- Clearly defining the structure to be measured
- Creating the item pool,
- Determining the measurement method,
- Incorporating validity criteria,
- Administering the scale to the sample group,
- Evaluating items,
- Optimizing the scale's length.

### Population and sample of the research

In line with the study's purpose and objectives, the focus group consisted of individuals aged 18 and above within the community. The convenience sampling method was employed for sample selection. Data were collected using an online survey conducted through Google Forms. At the outset of the survey, it has been explicitly stated that the purpose of the study and the voluntary nature of participation are established principles. Furthermore, it has been clarified that participants can proceed with the survey only after being provided with information and examining and consenting to it. Consequently, it is assumed that participants engaged in the study have read and affirmed this information.

The data collection process took place in 4 stages between April and October 2021. Several approaches can be used to select an appropriate sample size for a pilot study. According to Evci and Aylar (2017), 5% of the target audience can exhibit similar characteristics to those of the remainder of the target audience [25], and so Şeker and Gençdoğan (2006) suggested that a pilot study can be conducted by reference to 30 to 50 individuals who can represent a sample of the target audience for the scale under development [26]. Therefore, the pilot study was conducted by reference to 30 individuals. Regarding the sample size for factor analysis, it is important to obtain a sample size of 5 to 10 times the number of expressions included in the scale [27]. In the second stage, we plan to apply the 44-item scale to 440 people across Turkey. To perform the Exploratory Factor Analysis (EFA), 571 participants were reached; however, due to incorrect responses to the control question, 470 participants were retained, and the 22-item scale in the third stage was applied to 272 participants to perform the Confirmatory Factor Analysis (CFA) and ensure criterion validity.

### Data collection tool

In the study, the “Data Management in the Digital Health Environment Scale” developed by the researcher, “Norman and Skinner's E-Health Literacy Scale”, which was adapted to Turkish by Gencer (2017), the "Mobile Health and Personal Health Record Management Scale" developed by Arslan and Demir (2017) and a sociodemographic information form were used [28, 29]. The Data Management in the Digital Health Environment Scale consisted of the following 5 factors:

Benefit and satisfaction: Digital health applications allow individuals to obtain easy access to health services, to follow their health information and disease status, and to communicate with physicians more effectively. The items contained in this subscale measure the satisfaction of individuals with the digital health environment by focusing on these situations.

Security: The data stored in the digital health environment require high levels of protection. These data contain various important information about the health and disease status of individuals. This subscale is aimed at measuring the security perceptions of individuals regarding the data in question.

Sharing: Individuals have various responsibilities with regard to ensuring the security of the data retained in the digital health environment. Individuals’ unconscious sharing of information about their health status, especially on the internet and in the social media environment, entails that a great deal of data about individuals are generated. Access to these data by unauthorized persons leads to undesirable results. This subscale focuses on individuals’ sharing of their health-related status in digital environments.

Privacy: The data retained in the digital health environment include confidential information about the private lives of individuals. Unauthorized access to this information causes a violation of privacy. People must be aware of the importance of the data retained in the digital health environment, what their legal rights are, and how they should behave in case of privacy violations. This subscale focuses on individuals' perceptions of privacy regarding the digital health environment.

Use: Digital health technologies are generally web-supported. They offer an easy-to-use experience through digital health technologies, the internet and mobile technologies. Individuals are increasingly inclined to use digital health technologies to support their health status. This subscale aims to measure the tendency of individuals to use digital health applications.

### Mobile health and personal health record management scale

The Mobile Health and Personal Health Record Management Scale is a measurement tool developed by Arslan and Demir (2017) within the scope of the study "University Students Views on Mobile Health and Personal Health Record Management". This scale measures individuals’ views on m-health and personal health record management. The measure is scored on a 5-point Likert-type scale (strongly disagree, disagree, partially agree, agree, strongly agree). It consists of 31 questions and 4 subscales. The Cronbach's alpha coefficient of the scale was found to be 0.965 [29].

### Electronic Health (e-Health) literacy scale

The Turkish validity and reliability of the scale developed by Norman and Skinner (2015) were investigated by Gencer (2017) [28, 30]. This scale was developed to measure individuals' perceptions of the use of information technologies with regard to health-related issues and to help identify the harmony between e-health and individuals. The measure, which consists of 8 items and a single dimension, is scored on a 5-point Likert scale (strongly disagree, disagree, neutral, agree, strongly agree). As a result of the validity and reliability calculations, an 8-item scale was obtained. The factor structures of the scale were found to be valid. It was determined that the internal consistency coefficient of the scale was 0.863, and the test–retest reliability was 0.886 [28].

### Establishing the item pool

The first step in developing a scale is to determine the purpose and create an item pool [24]. As part of the creation of the item pool, a literature review was conducted. In such a literature review, drawing beneficial information from previously developed scales, collecting expert opinions, asking the target audience open-ended questions, and choosing the items that the researcher views as appropriate are the most commonly used methods [31]. Since the correlations among the items are not known during the item pooling phase, the large number of items represents a precaution against low internal consistency [24]. A draft scale containing 49 items in total was created by collecting the opinions of expert academicians. While creating these items, the dimensions of confidentiality, security, privacy, benefit and satisfaction, use and sharing, which were identified through the literature review, were taken into consideration, and linguistic and structural guidelines were followed [32].

### Obtaining expert opinions

According to DeVellis (2021), the review of an item pool created by experts can confirm or invalidate the definitions of the structure to be measured [24]. In this process, experts who have good knowledge of the structure to be examined in the scale examine the statements included in the item pool with a focus on their conceptual structure. Although there is no restriction on the number of experts, at least three experts should be included according to the Royal Winds or Society for Nursing Research [31].

To determine the relevance of the question items created based on the literature review and to ensure content validity, the draft scale form was submitted to a total of 3 experts, including 2 academicians who are experts in the field of health management and 1 academician who is an expert in the Turkish language, to determine its suitability in terms of language.

An expert evaluation form was prepared to enable the experts to evaluate the items. According to the form, the experts were asked to respond "Not at all Appropriate," "Partly Appropriate," or "Appropriate" with regard to the degree of conformity exhibited by the items. According to the feedback obtained from the experts, no items were eliminated; however, questions related to the scale were finalized by making corresponding corrections.

### Collection of pilot study data

After collecting these expert opinions, the draft scale form, which was finalized by making corrections to the items, was applied to the sample group [33]. As a result of the pilot study, expression errors in the items were corrected [31]. The sample determined in the pilot study should represent the target audience [34].

Thirty people with similar characteristics to those of the sample participated in the pilot test, and the draft scale items were delivered online. The draft scale items were scored on a 5-point Likert-type scale and were graded as 1 "Strongly Disagree", 2 "Disagree", 3 "Neutral, 4 "Agree", or 5 "Strongly Agree". At the end of the online scale form, a separate question was asked that enabled the participants to express their thoughts and suggestions about the scale expressions (e.g., indicating questions with the same meaning, questions that were difficult to understand, and meaningless questions). According to the feedback received from this pretest, the scale expressions were changed, and questions that had the same meaning or were incoherent were eliminated. As a result, the final form to be applied to the original sample group was developed.

### Construct validity

In scale development studies, construct validity enables researchers to explain the results of the scale and identify the item that is related to a given result [35]. Two methods that are frequently used to test construct validity are hypothesis testing and factor analysis [36]. The method used in this study to ensure construct validity was factor analysis. Factor analysis identifies the extent to which the measurement tool explains and validates the structure to be measured. "EFA" is used when the factor structure of the scale is to be revealed, and "CFA" is used to confirm the factor structure [36]. Within the scope of this study, both EFA and CFA were conducted to ensure construct validity.

Prior to the EFA, the Kaiser‒Meyer‒Olkin (KMO) and Bartlett’s sphericity values were first examined to evaluate the suitability of the dataset for factor analysis. According to Field (2009), a dataset with a KMO value less than 0.50 cannot be factored in. This value indicates that the sample size is adequate for factor analysis. This significant test value indicates that the scale can consist of multiple factors [37, 38]. The slope graph is used to determine the number of factors. It should be noted that it is appropriate to select as many factors as the number of points at which the slope transitions to a horizontal shift [37, 39, 40].

If an item was included in two factors simultaneously and there was a difference of 0.100 or less between the factor loadings of the factors in which it was included, that item was identified as an overlapping item, and it has been noted that it is appropriate to exclude items with a factor loading of less than 0.300 [37, 39].

According to Güriş and Astar (2015), the variance value of an item should be 0.40 or higher [41]. In cases in which this value is below 0.40, it is recommended to remove the item from the scale. Accordingly, the KMO value, slope graph, factor loadings and variance values were checked.

Collecting the variables observed on a scale with more than one factor is defined as first-level CFA. In this model, items with similar variances were collected in the same factor [42]. CFA was conducted to establish a relationship between the observed variables (scale items) and the latent variables (factors) [43]. In this context, first-level CFA was performed. High factor loadings, low error variances, and factor correlations of less than 0.85 are among the characteristics of a suitable measurement tool. If the factor correlations exceed 0.85, model fit can be achieved with fewer factors than the number of factors identified in the structure [44]. Factor loadings should be 0.30 or higher [39].

After the PATH diagram is drawn, the t values of the items should be examined. If the t value exceeds 1.96, the item is considered to be significant at the 0.05 level; if the value exceeds 2.56, it is considered to be significant at the 0.01 level. Nonsignificant values should be removed from the scale [44–47].

At least three first-level factors are required to perform second-level CFA [39, 48]. Therefore, second-level CFA was applied within the scope of the study. Within the scope of the study, factor loadings, factor correlations and t values were examined.

### Similar scale validity

Criterion-based validity indicates that the item or scale is associated with some criteria or assumed standards [24]. Criterion validity is determined by evaluating the correlation scores of the scale with other measurement results related to the structure measured by the scale [36]. Accordingly, “Norman and Skinner’s E-Health Literacy Scale,” which was adapted to Turkish by Gencer (2017) and studied in terms of its validity and reliability to ensure criterion validity in the study, as well as the scale developed within the scope of the study “University Students’ Opinions on Mobile Health and Personal Health Record Management” by Arslan and Demir (2017) were used in this study [28, 29].

### Reliability analysis

Within the scope of the study, two methods, i.e., consistency and stability, were used to ensure the reliability of the scale. To measure the internal consistency of a scale, a value between 0 and 1 should be achieved. Internal consistency indicates the extent to which all items measure the same concept or structure, thus establishing the relationship between the items [49]. Cronbach’s alpha coefficient was used to determine internal consistency.

The test–retest method was used to test the stability of the scale. According to this method, the similarity ratio of the test scores obtained from applying the same test to the same sample twice provides the reliability ratio [26]. The scale developed within the scope of the study was applied twice to a group of 40 people with an interval of one month.

## Results

According to Table 1, 73.4% of the participants were female, and 26.6% were male. A total of 24.3% of the participants were aged between 18–22, 25.3% were aged between 23–27, 16.2% were aged between 28–32, 15.5% were aged between 33–37, 10.6% were aged between 38–42, and 8.1% were aged 42 or older. Regarding the participants’ marital status, 54% were married, and 46% were single. A total of 29.4% were homemakers, 23.8% were students, 13.2% were employed, 13.6% were civil servants, 13.8% were tradesman/self-employed, 1.1% were retired, and 5.1% were unemployed. Regarding their educational status, 30.2% of the participants were primary school graduates, 30.2% were high school graduates, 20% held associate degrees, 35.3% held bachelor’s degrees, and 4.9% held postgraduate degrees. The perceived monthly income of 40% of participants was low, that of 58.7% was at a medium level, and that of 1.3% was at a high level. Of the participants in the research, 98.9% used social media, while 1.1% did not use social media. The Kaiser‒Meyer‒Olkin (KMO) value and the Bartlett’s sphericity value are shown in Table 2.

**Table 1:** Frequency and percentage distributions of the participants in the Exploratory Factor Analysis in terms of demographic information

Variable	Subvariable	n	%
Gender	Female	345	73.4
	Male	125	26.6
Age	18–22	114	24.3
	23–27	119	25.3
	28–32	76	16.2
	33–37	73	15.5
	38–42	50	10.6
	42 +	38	8.1
Marital status	Married	254	54.0
	Single	216	46.0
Occupation	Homemaker	138	29.4
	Student	112	23.8
	Employee	62	13.2
	Officer	64	13.6
	Craftsman/Self-Employed	65	13.8
	Retired	5	1.1
	Unemployed	24	5.1
Educational status	Primary Education	45	9.6
	High School	142	30.2
	Associate Degree	94	20.0
	Bachelor’s Degree	166	35.3
	Postgraduate	23	4.9
Perceived income status	Low	188	40.0
	Middle	276	58.7
	High	6	1.3
Social media use	Yes	465	98.9
	No	5	1.1

**Table 2:** Explanatory factor analysis of the Kaiser‒Meyer‒Olkin and bartlett's test results regarding the Data Management in the Digital Health Environment Scale

Kaiser-Mayer-Olkin (KMO)		.857
Bartlett’s Test	x^2^	3912.951
	df	231
	p	.000

**Fig. 1:**
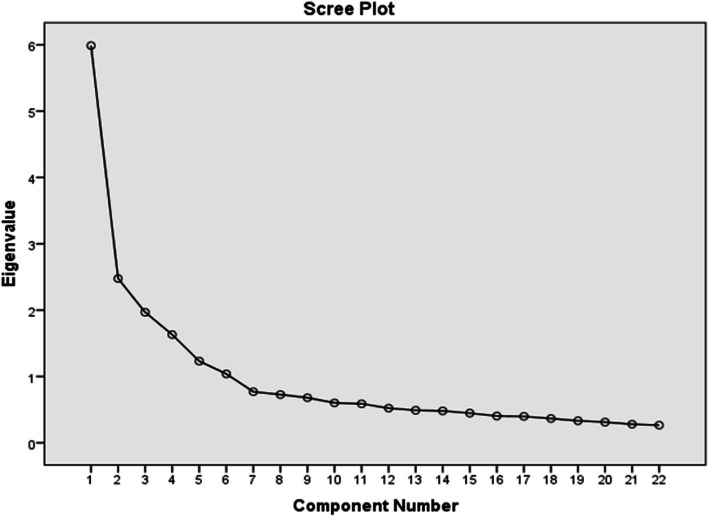
Slope Graph of the Explanatory Factor Analysis for the Data Management in the Digital Health Environment Scale

According to Table 2, the KMO value was 0.857 [50]. Bartlett's sphericity value was significant (*p* < 0.05). The slope graph is illustrated in Fig. 1.

According to Fig. 1, the graph starts to exhibit a linear structure from points 5 and 6. In addition, five factors feature one or more eigenvalues [31]. Therefore, five factors were selected for the factor analysis. The factor loadings, explained variance values and common factor variance are shown in Table 3.

**Table 3:** Explanatory factor analysis factor loadings for the Data Management in the Digital Health Environment scale

Items	Factor 1	Factor 2	Factor 3	Factor 4	Factor 5	Explained Variance (%)	Common Factor Variance
(16)* 32. I consider the development of digital health applications to be beneficial for the health system	.763					19.85	.630
(14)* 30. Thanks to my digital health records, my physician's ability to monitor my condition remotely increases my satisfaction with health services	.742						.615
(15)* 31. It is very beneficial for my doctor to access my digital health records and examine me using these data	.733						.566
(18)* 34. I can easily access the health information I want by using digital health applications	.710						.524
(12)* 28. My health records in digital media accelerated the process of receiving service from the health institution	.694						.510
(13)* 29. I think that my health information in the digital environment facilitates the work of authorized health personnel	.675						.491
(17)* 33. I recommend the use of digital health applications to my acquaintances	.667						.548
(19)* 35. I am not worried about downloading the mobile application of the Ministry of Health to my smart device regarding my health	.595						.417
(6)* 12. The authorities have taken all kinds of precautions to secure my health information in the digital environment		.852				14.18	.756
(4)* 9. I think that my digital health records are securely protected		.800					.726
(7)* 14. The legal regulations for protecting my health records in the digital environment are sufficient		.742					.609
(5)* 10. The legal system will protect me if my health records that are retained in digital media are accessed by third parties without permission		741					.610
(8)* 19. From the moment I enter the Health Institution, the health care personnel who access my data in the digital environment take care of my privacy		.571					.509
(10)* 26. I can share my health status in health-related groups of which I am a member on the internet			.828			9.71	.729
(9)* 25. I can share my personal information in health-related groups on the internet of which I am a member			.803				.687
(11)* 27. I can share my personal information while shopping on health-related e-commerce sites on the internet			.756				.586
(2)* 6. My health records in digital media contain critical information regarding my personal privacy				.844		8.63	.719
(1)* 5. It is my legal right to request that my health information in digital media be kept confidential				.833			.715
(3)* 7. If I notice a confidentiality gap regarding my health records in the digital environment, I immediately contact the authorities of the relevant institution				.653			.530
(21)* 41. I like to follow the developments in digital health applications closely					.780	8.06	.695
(22)* 44. I shop online at health-related websites					.670		.530
(20)* 40. I do not avoid being a member of health-related groups on the internet					.669		.592

^*^Item number in the final version of the scale

In Table 3, the scales with a boarding or factor loading below 0.300, i.e., Items 1, 2, 3, 4, 8, 11, 13, 15, 16, 17, 18, 20, 21, 22, 23, 24, 36, 37, 38, 39, 42 and 43, were removed from the scale. After these items were removed from the scale, EFA was conducted once again. Table 3 shows that the scale consists of 5 factors. Factor 1 was named “benefit and satisfaction”, Factor 2 “security”, Factor 3 “sharing”, Factor 4 “privacy", and Factor 5 "use". The total variance explained by the scale was 60.43%. An important criterion in factor analysis is that the explained variance exceeds 50% of the total variance. If the factor structure formed based on this analysis explains less than half of the total variance, it is not possible to suggest that the results are representative [51]. The value of 60.43% emerged from the analysis, i.e., in excess of 60% [31], which is accepted as the lower limit in the social sciences. Common factor variance values related to the scale items varied between 0.417 and 0.756.

### Confirmatory factor analysis of the data management in the digital health environment scale

**Fig. 2:**
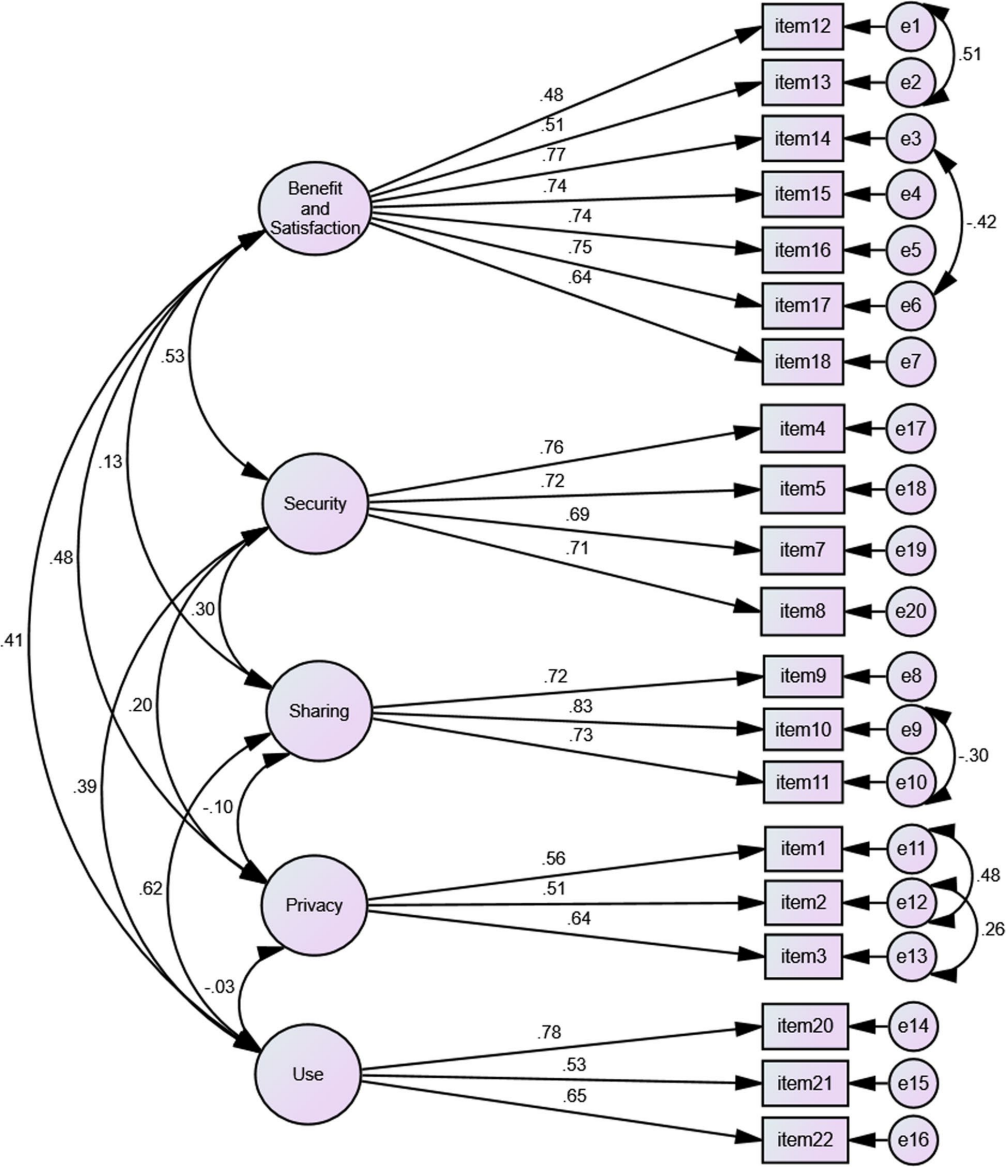
First-Level Confirmatory Factor Analysis Path (PATH) Analysis of the Data Management in the Digital Health Environment Scale

This section includes the CFA results of the Data Management in the Digital Health Environment Scale. After identifying the scale's factor structure using EFA, CFA was performed to identify the quality of this factor structure, the general structure of the scale, and the extent to which the scale explained the Data Management in the Digital Health Environment Scale. Figure 2 shows the first-level factor analysis results of the scale.

According to Fig. 2, the correlation between the factors is below 0.85. In the first-level CFA chart of the Data Management in the Digital Health Environment Scale, two items of the 22-item scale are not included (Item 6 and Item 19). These items were excluded from the scale due to their low regression coefficient. The regression coefficients for the remaining items ranged from 0.48 to 0.83.

The standard error and t values of the first-level factor analysis are shown in Table 4.

**Table 4:** Standard Error and t Values for the first-level confirmatory factor analysis of the Data Management in the digital health environment scale

Items	Std. Error	t	*p*
f1	.149	3.956	***
f2	.410	6.679	***
f3	.578	5.678	***
f4	.113	3.247	***
f5	.546	6.327	***
e1	.508	11.239	***
e2	.385	11.162	***
e3	.192	8.570	***
e4	.264	9.873	***
e5	.223	9.836	***
e6	.299	8.853	***
e7	.251	10.671	***
e8	.532	7.208	***
e9	.361	3.727	***
e10	.521	5.796	***
e11	.253	7.420	***
e12	.204	7.199	***
e13	.295	5.525	***
e14	.355	6.175	***
e15	.642	10.322	***
e16	.669	8.922	***
e17	.304	8.207	***
e18	.476	8.928	***
e19	.383	9.395	***
e20	.330	9.106	***

^***^p < 0.01

**Fig. 3:**
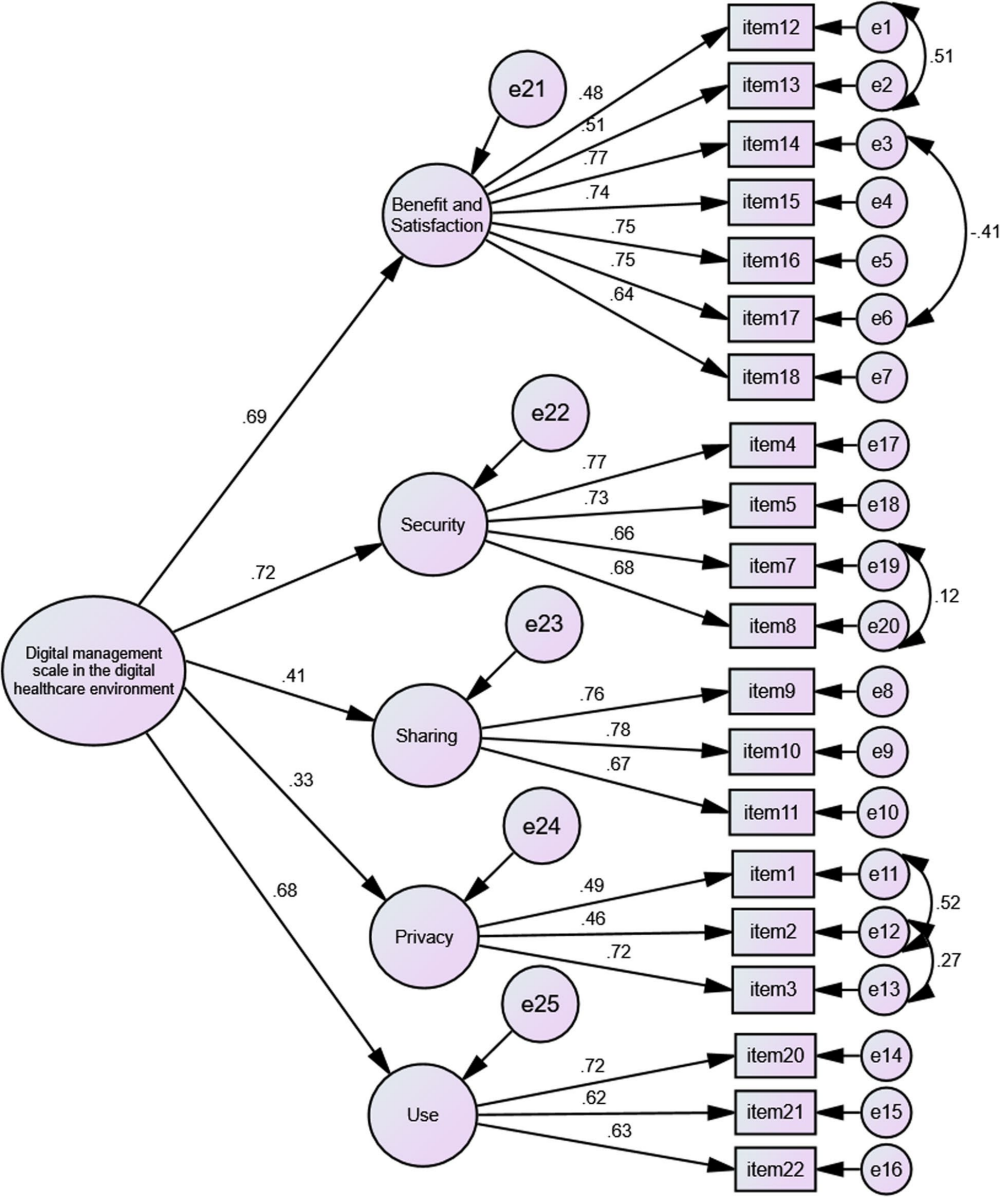
Second-Level Confirmatory Factor Analysis Path (PATH) Analysis of the Data Management in the Digital Health Environment Scale

According to Table 4, the variance values for all items and subscales are significant. Figure 3 shows the second level factor analysis results of the scale.

According to Fig. 3, the regression coefficients for the items of the 20-item scale remaining after the first-level CFA vary between 0.48 and 0.78. The factor loadings of the overall score on the subscales vary between 0.33 and 0.72. The dimension that affects data management in the digital health environment most strongly is "security", while the least effective subscale is "privacy”.

The standard error and t values of the second-level factor analysis are shown in Table 5.

**Table 5:** Standard error and t Values for the second-level confirmatory factor analysis of the data management in digital health environment scale

Items	Std. Error	t	*p*
Total Score	.023	3.152	.002
e1	.023	3.497	***
e2	.046	4.356	***
e3	.090	5.965	***
e4	.039	2.038	**
e5	.063	4.012	***
e6	.045	11.212	***
e7	.035	11.148	***
e8	.023	8.463	***
e9	.027	9.808	***
e10	.023	9.743	***
e11	.034	8.731	***
e12	.024	10.661	***
e13	.067	6.911	***
e14	.072	6.405	***
e15	.069	9.031	***
e16	.047	5.945	***
e17	.044	4.894	***
e18	.118	2.024	**
e19	.064	6.824	***
e20	.062	8.782	***
e21	.081	8.731	***
e22	.038	7.641	***
e23	.054	8.463	***
e24	.044	9.089	***
e25	.040	8.949	***

^**^p < 0.05; ***p < 0.01

According to Table 5, the variance values ​​for Items e4 and e18 are significant at *p* < 0.05, and the variance values ​​for the other items and subscales are significant at *p* < 0.01.

The fit indices for the first/second-level factor analysis are shown in Table 6.

**Table 6:** Evaluation of fit indices related to the first-level-second-level confirmatory factor analysis of the Data Management in the Digital Health Environment Scale

Fit Indices	Standard Value (Good Fit)	Acceptable Fit	Measurement Value (First Level)	Measurement Value (Second Level)
chi-square/sd (X2/sd)	≤ 2	≤ 5	1.980	2.396
Root Mean Square Error of Approximation (RMSEA)	≤ .05	≤ .09	.060	.072
Comparative Fit Index (CFI)	≥ .95	≥ .90	.924	.936
Goodness-of-Fit Index (GFI)	≥ .95	≥ .90	.900	.948
Adjusted Goodness-of-Fit Index (AGFI)	≥ .90	≥ .85	.864	.877
Root Mean Square Residuals (RMR)	≤ .05	≤ .08	.050	.068
Standardized Root Mean Square Residual (SRMR)	≤ .05	≤ .08	.069	.089

References [40, 45, 46, 52–54]:

According to Table 6, the values obtained from the first/second-level confirmatory factor analysis of the fit values (Chi-square/sd = 1.980/2.396; RMSEA = 0.060/0.072; CFI = 0.924/0.936; GFI = 0.900/0.948; AGFI = 0.864/0.877; RMR = 0.050/0.068; SRMR = 0.069/0.089) indicate an acceptable level of agreement, and the CFA results indicate that the construct validity of the scale is appropriate.

### Examination of similar scale validity of the data management in the digital health environment scale

Table 7 shows the correlation results between the scales.

**Table 7:** Pearson’s correlation analysis results for the relationships among the mobile health and personal health record management scale, the e-health literacy scale and the data management in the digital health environment scale

		**Benefit and Satisfaction**	**Security**	**Sharing**	**Privacy**	**Use**	**Data Management in the Digital Health Environment Scale Overall Score**
Accessibility	r	.636^**^	.312^**^	-0.033	.322^**^	.219^**^	.500^**^
	p	.000	.000	.593	.000	.000	.000
Benefiting	r	.709^**^	.375^**^	.124^*^	.303^**^	.381^**^	.638^**^
	p	.000	.000	.041	.000	.000	.000
Reliability	r	.585^**^	.678^**^	.187^**^	.116	.387^**^	.686^**^
	p	.000	.000	.002	.057	.000	.000
Availability	r	.551^**^	.355^**^	.034	.242^**^	.203^**^	.480^**^
	p	.000	.000	.581	.000	.001	.000
Mobile Health and Personal Health Record Management Scale Overall Score	r	.733^**^	.486^**^	.082	.300^**^	.346^**^	.669^**^
	p	.000	.000	.179	.000	.000	.000
E-Health Literacy Scale Overall Score	r	.348^**^	.267^**^	.069	.210^**^	.273^**^	.378^**^
	p	.000	.000	.257	.000	.000	.000

^**^p < 0.01; *p < 0.05

According to Table 7, there are no significant relationships among sharing, which is the focus of one subscale of the Data Management in the Digital Health Environment Scale, and the accessibility and usability subscale and the total score, which are subscales of the Mobile Health and Personal Health Record Management Scale. There were no significant relationships with regard to the total literacy scale score (*p* > 0.05). There were positive and significant relationships between the sharing subscale of the Data Management in the Digital Health Environment Scale and perceptions of reliability and benefit, which are subscales of the Mobile Health and Personal Health Record Management Scale (*p* < 0.05). There were positive and significant relationships among utility and satisfaction, security, privacy, usage, the overall score in the Data Management in the Digital Health Environment Scale, the overall score of the Mobile Health and Personal Health Record Management Scale, and the e-Health Literacy Scale (*p* < 0.05). These findings indicate that similar scale validity was achieved since positive and significant relationships were observed among the majority of the subscales.

### Reliability analysis

Cronbach's alpha coefficient was used to determine internal consistency. Table 8 shows the results in terms of Cronbach's alpha coefficient.

**Table 8:** Reliability analysis results regarding the data management in the digital health environment scale

Subscale	Number of items	Cronbach’s alpha	Scale items
Benefit and satisfaction	8	.865	12,13,14,15,16,17,18,19*
Security	5	.843	4,5,6*,7,8
Sharing	3	.758	9,10,11
Privacy	3	.697	1,2,3
Use	3	.631	20,21,22
Total score	22	.856	All items except 6 and 19

^*^ Items not used in scoring since they were removed after confirmatory factor analysis

According to Table 8, the sharing, privacy, and usage subscales of the Data Management in the Digital Health Environment Scale are moderately reliable. In contrast, the utility and satisfaction, security subscales, and overall scale score are highly reliable [42]. Therefore, the scale's reliability is ensured.

The test–retest method analysis results are shown in Table 9.

**Table 9:** Comparison of test–retest results of the items included in the Data Management in the Digital Health Environment Scale

Subscale	Group	n	X	Ss	t	p
Benefit and satisfaction	Pretest	40	3.56	.632	.303	.763
	Final Test	40	3.54	.626		
Security	Pretest	40	4.44	.593	1.688	.099
	Final Test	40	4.34	.556		
Sharing	Pretest	40	3.40	.970	-0.429	.671
	Final Test	40	3.45	.859		
Privacy	Pretest	40	4.54	.470	-0.121	.905
	Final Test	40	4.55	.358		
Use	Pretest	40	3.18	.946	.525	.602
	Final Test	40	3.14	.954		
Total Score	Pretest	40	3.80	.521	.648	.648
	Final Test	40	3.78	.442		

Twenty items included in the final version of the scale used within the scope of this research were applied to the group of 40 people twice, with an interval of one month between the applications. There were no significant differences between the responses of the participants to the subscales and the total score for both applications (*p* > 0.05). The absence of significant differences indicates that the responses provided by the respondents were consistent across different times and thus that the questions were understood in similar ways at these different times. Therefore, the measurement ranges of the scale questions are consistent. Table 10 shows the correlation results among the subdimensions.

**Table 10:** Investigation of the relationship between the test–retest results of the subscales included in the Data Management in the Digital Health Environment Scale

	**Benefit and satisfaction**	**Security**	**sharing**	**Privacy**	**Use**	**Total Score**
Benefit and satisfaction	.888**					
Security		.789**				
Sharing			.681**			
Privacy				.470**		
Use					.861**	
Total Score						.909**

*n* = 40

According to Table 10, the correlation coefficient between the first and second tests regarding the subscales is 0.888 for the utility and satisfaction subscale; 0.789 for the security subscale; 0.681 for the sharing subscale, 0.470 for the privacy subscale, and 0.861 for the usage subscale. The total score was found to be 0.909. According to these values, the subscales and the overall score are related across the two tests, and a high level of reliability is provided for the overall scale.

## Discussion and conclusion

In accordance with the relevant literature, the researcher planned to develop a measurement tool for data management in the digital health environment to compensate for the limited number of studies measuring individuals' perceptions of their data in the digital health environment. This measurement tool can be used in studies on this topic.

The Data Management in the Digital Health Environment Scale provided five factors that explain 60.43% of the total variance. The factor loading values ​​of the scale items ranged from 0.571 to 0.852. The subscales were named privacy, security, usage, sharing, benefit, and satisfaction. First- and second-level CFA were conducted to test the accuracy of the structure formed as a result of the EFA. The fit indices calculated for the model were Chi-square/sd = 1,980 for the first-level CFA; RMSEA = 0.060; CFI = 0.924; GFI = 0.900; AGFI = 0.864; RMR = 0.050; SRMR = 0.069; Chi-square/sd = 2.396 for second-order CFA; RMSEA = 0.072; CFI = 0.936; GFI = 0.948; AGFI = 0.877; RMR = 0.068; SRMR = 0.089. These values ​​indicate that the fit indices are within an acceptable range. To test the criterion validity, Norman and Skinner's E-Health Literacy Scale (Gencer, 2017) and the measurement tool developed by Arslan and Demir (2017) as part of the study "University Students' Views on Mobile Health and Personal Health Record Management" were used [28, 29]. The results of the analysis indicated positive and significant relationships (*p* < 0.05) between the measurement tools and the Data Management in the Digital Health Environment Scale. After the scale's construct validity was ensured, Cronbach's alpha coefficient was calculated to determine internal consistency, and reliability was ensured by using the test–retest method. Cronbach's alpha coefficient was calculated as 0.856. As a result of the test–retest, the correlation coefficient was calculated as 0.909, and it was thus concluded that there were no significant differences between the participants' responses across the two time points (*p* > 0.05). The findings of this study demonstrate the validity and reliability of the Data Management in Digital Health Environment Scale, as evidenced by its strong factor structure, positive correlations with related measurement tools, and high internal consistency. These results support the scale's suitability for assessing data management practices in the digital health domain, providing valuable insights for future research and practical applications.

According to the relevant literature, many studies have investigated the topic of digital health. The fields of study related to digital health generally emphasize health information technologies, e-health, and m-health. While the existing literature on digital health has explored various aspects of this field, there still needs to be a notable gap in scale development and adaptation. This study addresses this gap by introducing the Data Management in the Digital Health Environment Scale, contributing to the broader understanding of digital health measurement tools.

In the study conducted by Wilson and Lankton (2004) on the e-health acceptance of patients, a reliability result of over 0.900 was achieved, and the developed model exhibited acceptable compliance values [55]. The fit indices (all GFI, CFI, NFI, and IFI values) for the structural model of the scale used in the study conducted by Deng et al. (2018) on m-health care adoption were greater than 0.900. All fit indices of the research model were above the normal mean acceptance level [56]. In a study on the adoption of m-health services by elderly users, the reliability values for the subdimensions varied between 0.888 and 0.932. The results indicated that the factors explained 81.5% of the variance [57]. In a study on the adaptation of the electronic health literacy scale to Chinese culture, Cronbach's coefficient was found to be 0.907. The consistency coefficient of test–retest reliability was 0.691. Three factors were obtained by EFA, and these three factors accounted for 90.84% of the total variance. Factors loaded on 19 items ranged from 0.806 to 0.944. As a result of DFA, it was concluded that the model had good fit values (NFI = 0.979, RFI = 0.955, IFI = 0.987, TLI = 0.972, CFI = 0.987, RMSEA = 0.070, CMIN/DF = 2.586). The KMO value was found to be 0.850, and it was concluded that Bartlett’s test of sphericity was significant (*p* < 0.01) [58]. In the study conducted by Octavius and Antonio (2021) on the intention to accept m-health apps, ten participants (five men and five women) using m-health apps were pretested, and then a pilot study including thirty participants was conducted to further develop the study [59]. In a study on the development of the e-health literacy scale, a total of 89 individuals (14–24 years old) were selected as participants in the pilot study. The factor loadings of the 8 items that emerged as a result of the analyses ranged from 0.60 to 0.84. According to the test–retest results regarding the scale, the stability of the scale over time is good [30]. However, the literature has provided few measurement tools for individuals' perceptions of the digital health environment. Therefore, this study contributes to the literature in this respect. In summary, while previous research has yielded valuable insights into the acceptance and assessment of e-health and m-health technologies, there needs to be measurement tools focusing on individuals' perceptions of the digital health environment. This study fills that gap by introducing Data Management in the Digital Health Environment Scale, which is expected to enhance our understanding of the evolving digital health landscape and facilitate further research in this critical area.

Consequently, the statistical analysis results of the scale developed within the scope of this study indicate that the scale exhibits similar characteristics to those reported by previous studies. Focusing on the opportunities and threats associated with digital health, a valid and reliable scale was developed to measure individuals' perceptions of use and sharing in the digital health environment, their perceptions of security and privacy regarding their data that are stored in the digital environment, and their satisfaction with these systems within the scope of the study. Developing a scale to determine the attitudes of individuals towards their data in the personal digital health environment; Organizing personal health information, increasing patient participation, providing personal health follow-up, facilitating drug management, helping health decisions, supporting being a conscious consumer, determining personal health goals and reaching these goals and ensuring data privacy and security are important in determining behaviors. In addition, determining the knowledge and attitudes of individuals towards the digital health environment can contribute to improving health services, increasing the quality of care for patients, encouraging research and innovation, protecting data security and privacy, increasing cost-effectiveness, and better management of health data. Therefore, it is anticipated that the present study will contribute to the literature. It is recommended that future research be conducted to identify the perceptions of individuals regarding the digital health environment by considering the dimensions of the scale one by one. The scale developed in the current study should be used in more studies, and its validity should be tested in larger populations. The usability of the scale in different cultures and languages should also be evaluated. This way, an international comparison of individuals' attitudes toward the digital health environment can be achieved. Additionally, the scale can be compared with other measurement tools to assess the factors affecting individual attitudes toward the digital health environment. Studies can be conducted to evaluate the impact of individuals' attitudes toward the digital health environment on their health behaviors and outcomes. Furthermore, research that investigates the relationship between individuals' education levels and their attitudes toward the digital health environment can be conducted. This could contribute to the development of better educational programs and awareness campaigns.

### Limitations of the research

The limitations of this research lie in the fact that its results are limited to the answers provided by the individuals who participated in the research and the online administration of the survey.

### Glossary of terms

- Digitalization: The process of transforming information into digital format.
- Digital Transformation: Integrating digital technologies into an organization or an all industry.
- eHealth: The use of digital technologies for healthcare services.
- Privacy: Protection of personal and sensitive health data from unauthorized access.
- Security: Safeguarding health data from breaches, cyberattacks, and unauthorized access.
- Data Management: The collection, storage, and organization of health-related information in digital form.
- Health Informatics: The field concerned with information and communication technologies in healthcare services.
- Mobile Health (mHealth): Utilizing mobile devices for various health purposes.
- Electronic Health Record (EHR): The digital version of paper-based medical records.
- Digital Health Environment: The environment where information and resources obtained through the delivery of healthcare services are stored, shared, and managed in digital platforms.
- Data Management in the Digital Health Environment: The ability of individuals to manage their health information and data in the digital health environment, ensuring the privacy and security of this data, as well as perceptions of privacy, security, benefits, satisfaction, sharing, and usage related to the digital health environment.
- Health Literacy: The skill of an individual in understanding and utilizing health information.

## Data Availability

The datasets used and/or analysed as part of the current study are available from the corresponding author upon reasonable request.
